# P-1893. An Interactive, Case-Based Microbiology Laboratory Session for First-Year Medical Students

**DOI:** 10.1093/ofid/ofaf695.2062

**Published:** 2026-01-11

**Authors:** Eric S Karney, Gabriella Smith, Julia Hankins, Robert Hamilton-Seth, Rachel Liesman, Mimi Precit, Jessica R Newman, Erica MacKenzie

**Affiliations:** University of Kansas School of Medicine, Kansas City, KS; University of Kansas School of Medicine, Kansas City, KS; University of Kansas Health System, Kansas City, Kansas; University of Kansas Health System, Kansas City, Kansas; Medical College of Wisconsin, Milwaukee, Wisconsin; Providence Health and Services, Portland, Oregon; University of Kansas Medical Center, Fairway, Kansas; University of Kansas Health System, Kansas City, Kansas

## Abstract

**Background:**

Although in-person anatomy sessions continue to be a fundamental component of the medical school curriculum, laboratory-based microbiology sessions are increasingly replaced with virtual equivalents or removed altogether. In the integrated organ system-based curriculum at the University of Kansas School of Medicine, first-year medical students are introduced to medical microbiology primarily through classroom-based methods (e.g., lectures, flipped classes, and case-based collaborative learning).

**Figure:**
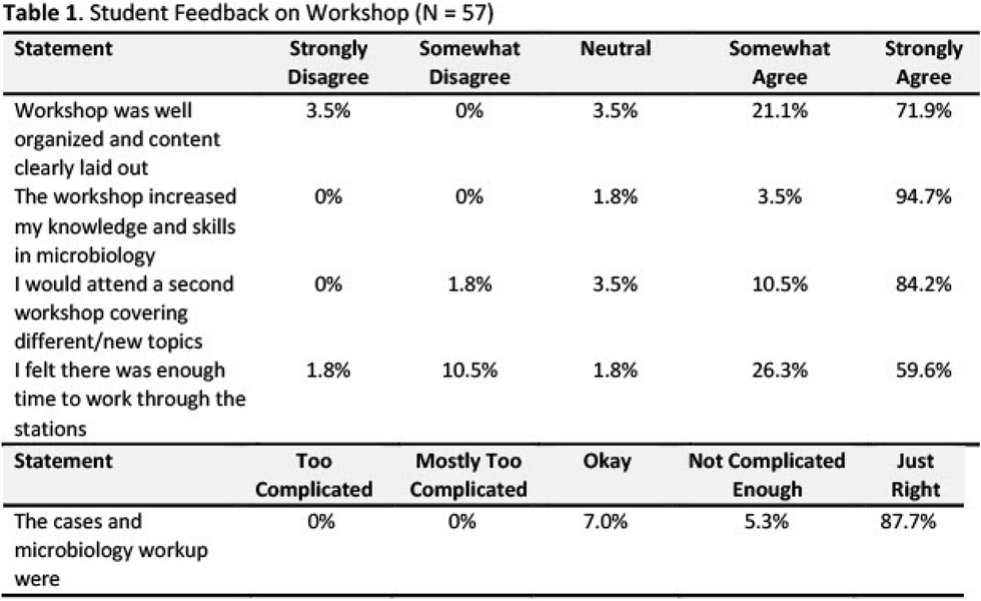

**Figure:**
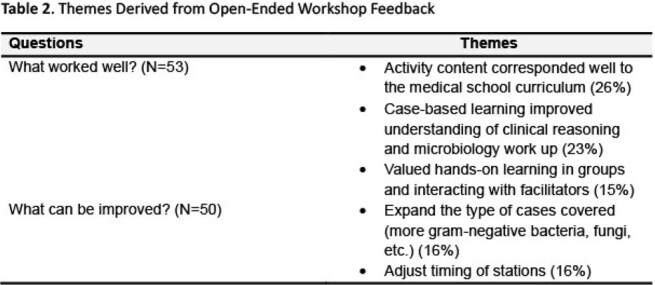

**Methods:**

An optional, extracurricular, “wet” laboratory session was developed to reinforce and build upon abstract microbiology concepts covered in a classroom setting. Students worked in small groups to solve short case studies involving bacterial and fungal pathogens. Cases required students to recall clinical presentations of common infections, interpret Gram stains, perform biochemical tests, and make a presumptive diagnosis.

**Results:**

This workshop has been successfully implemented for three consecutive years and has been well received by 57 students. In post-session evaluations, participants strongly agreed that the session enhanced their understanding of clinical microbiology concepts from their Infectious Disease lecture series and greatly appreciated the hands-on, case-based learning experience with facilitators.

**Conclusion:**

According to first-year students, interactive microbiology labs are a beneficial component of the pre-clerkship medical school curriculum. Future plans include implementing pre- and post-session assessments to better assess students’ knowledge acquisition and retention after laboratory-based sessions.

**Disclosures:**

All Authors: No reported disclosures

